# Impact of carbon dioxide concentrations on laboratory sensitivity of *Mycoplasma* species isolated from dairy cows

**DOI:** 10.1128/spectrum.00946-24

**Published:** 2024-08-20

**Authors:** Marit M. Biesheuvel, Kristen Kalbfleisch, Jeroen De Buck, Erik van Engelen, Gerdien van Schaik, Paolo Moroni, Gloria Gioia, Diego Nobrega, Gennerick J. Samera, Paul J. Adams, Jermaine R. Walcott, Herman W. Barkema

**Affiliations:** 1 Faculty of Veterinary Medicine, University of Calgary, Calgary, Alberta, Canada; 2 Royal GD, Deventer, The Netherlands; 3 Department of Population Health Sciences, Faculty of Veterinary Medicine, Utrecht University, Utrecht, The Netherlands; 4 Quality Milk Production Services, Animal Health Diagnostic Center, Cornell University, Ithaca, New York, USA; 5 Dipartimento Medicina Veterinaria e Scienze Animali, Universita' Degli Studi di Milano, Lodi, Italy; 6 Applied Genomic Centre, Kwantlen Polytechnic University, Surrey, British Columbia, Canada; MultiCare Health System, Tacoma, Washington, USA

**Keywords:** *Mycoplasma *spp., culture procedures, dairy cows, bacterial growth, carbon dioxide, laboratory sensitivity

## Abstract

Conventional *Mycoplasma* spp. diagnostics involve culture, often considered the gold standard in diagnostic test evaluation. However, culture protocols lack empirical derivation and primarily adhere to National Mastitis Council recommendations, tracing back to initial cultivation of *Mycoplasma bovis*. Despite a wide range of carbon dioxide (CO_2_) supplementation reported in literature, specific impacts of CO_2_ on *Mycoplasma* spp. growth remain unexplored. Our objective was to assess the effect of CO_2_ concentration on growth detection rates of 24 *Mycoplasma* spp. isolates from dairy cows. These isolates, mainly *M. bovis*, were incubated at 37°C in triplicate and three dilution ranges under three CO_2_ conditions: ambient air or 5% CO_2_ or 10% CO_2_. Bacterial growth was evaluated on incubation days 3, 5, 7, and 10. When cultured using ambient air, log_10_ cfu/mL was lower on days 3, 5, and 7 of incubation compared with isolates incubated in the recommended 5% or 10% CO_2_, with less variation observed in ambient air compared with 5% or 10% CO_2_. However, by 10 days of incubation, no differences in the detection of observable growth were noted among isolates incubated in ambient air, 5% CO_2_, or 10% CO_2_. Consequently, *Mycoplasma* spp. isolated from dairy cattle demonstrated growth after the recommended 7–10 days of culture, even in the absence of supplemental CO_2_. Given the expected concentration of *M. bovis* in (sub)clinical samples had similar concentrations to those used in our study, with the majority of isolates being *M. bovis*, we recommend expanding CO_2_ concentration ranges in *M. bovis* culture from 10% CO_2_ to ambient air when incubating for 10 days. However, the turnaround time could be shortened when incubating with supplemental CO_2_.

**IMPORTANCE:**

Current *Mycoplasma* spp. culture protocols lack empirical derivation concerning carbon dioxide (CO_2_) supplementation and are primarily based on the initial cultivation of *Mycoplasma bovis*. This study indicates that the suitable range for CO_2_ supplementation is broader than what is currently recommended by the National Mastitis Council for culturing within the specified 7–10 days. No differences in bacterial growth detection rates were observed among ambient air, 5% CO_2_, or 10% CO_2_ supplementation during the 7- and 10-day incubation intervals. These new insights provide evidence supporting the possibility of culturing *Mycoplasma* spp. under ambient air conditions in a laboratory setting.

## INTRODUCTION

*Mycoplasma bovis* was initially identified from a severe case of bovine mastitis in the USA in 1961 (1). Since then, *M. bovis* has spread globally through animal movements (2). Infections caused by *Mycoplasma* spp. can be diagnosed with conventional bacteriological culture, PCR, and ELISA. Culture is often considered the gold standard and used to validate diagnostic tests (3, 4). Currently, protocols for culturing *Mycoplasma* spp. in milk samples adhere to recommendations of the National Mastitis Council (NMC), based on the inaugural study in reference (1), wherein *M. bovis* was successfully cultured for the first time (5). Isolates were obtained after incubating in 10% carbon dioxide (CO_2_) for 5 days (1). However, subsequent studies revealed successful culturing using various CO_2_ concentrations, such as 5% CO_2_ (6, 7), candle jars with elevated CO_2_ concentrations (8), and as low as the CO_2_ present in ambient air (~0.4%) (9, 10).

Recently, it was suggested that the range of suitable CO_2_ concentrations and incubation periods may be broader than currently acknowledged (11). They reported that growth of bovine *Mycoplasma* isolates was not different when incubated in concentrations of 2.7%, 5%, or 10% CO_2_. Moreover, these authors concluded that 100% of the *M. bovis* laboratory isolates were detected after 3 days of incubation, regardless of CO_2_ concentration (11).

*Mycoplasma* spp. have been described as facultative (12), indicating that they are primarily aerobic but may switch to anaerobic pathways in the absence of oxygen (13). Therefore, *M. bovis* has been characterized as relatively insensitive to atmospheric conditions and can be successfully cultured under ambient conditions (13), suggesting that CO_2_ may not be essential for *Mycoplasma* growth and primary isolation. It was also highlighted in reference (11) that recommendations for CO_2_ concentrations lack empirical derivation. Considering that access to a CO_2_ incubator is sometimes limited and impractical, it becomes crucial to assess the impacts of CO_2_ concentration on *Mycoplasma* spp. growth.

To ascertain whether the current recommendations for culturing *Mycoplasma* spp. isolated from dairy cows should be revised, a comprehensive evaluation of various culture protocols under standardized circumstances is imperative. Our specific objectives were to (1) assess the detection rates of *Mycoplasma* spp. laboratory isolates collected from clinically and subclinically diseased dairy cows (mastitis, arthritis, and respiratory disease) after incubation under a range of CO_2_ concentrations, including ambient air, 5% CO_2_, or 10% CO_2_, and (2) compare the impacts of CO_2_ concentrations on growth rate at various incubation time points.

## MATERIALS AND METHODS

### Isolates

The study used 17 *Mycoplasma* laboratory isolates obtained from the Quality Milk Production Services, Animal Health Diagnostic Center, Cornell University, Ithaca, NY. DNA of each isolate was purified, and the identity was confirmed at Quality Milk Promotion Services through a multitarget PCR assay that enabled identifying *M. bovis* at the species level and discriminating *Mycoplasma* from *Acholeplasma* at the genus level (14). *Mycoplasma* isolates not identified as *M. bovis* were submitted for Sanger sequencing of PCR products. Freshly cultured isolates were then shipped in 15% glycerol and pleuropneumonia-like organisms (PPLO) broth to the University of Calgary. Upon arrival, isolates were immediately inoculated in Mycoplasma Broth (Cat#R102, Hardy Diagnostics, Santa Maria, CA) and incubated for 3 days at 37°C, 5% CO_2_, at which time glycerol stocks were created using a final concentration of 30% glycerol (15).

In addition to the 17 laboratory isolates, 28 *M*. *bovis* PCR-positive samples (swabs, tissues, milk, and fluids) were acquired from the Applied Genomic Centre at Kwantlen Polytechnic University (KPU) (Surrey, BC, Canada). These samples (swabs, tissues, milk, and fluids) had been stored frozen at −20°C from 1 month to >3 years before being shipped frozen overnight to the University of Calgary. Upon arrival, samples were thawed and cultured according to the NMC Guidelines for milk, the guidelines outlined in Clinical Veterinary Microbiology (CVM) (16) and procedures outlined in reference (17) (swabs, tissues, and fluids). Mycoplasma Broth (Cat# R102, Hardy Diagnostics, Santa Maria, CA) and PPLO Agar produced in house following a formulation used by Royal GD (Deventer, the Netherlands, Supplementary Materials A) were used for culturing (paragraph 2.2). *M. bovis* was isolated from seven samples and used for this experiment. Identification of the seven pure *M. bovis* cultures was confirmed by Sanger sequencing and Illumina WGS at KPU. Identification by 16S was performed using primers 27F and 1492R (18). Additional custom primers (Integrated DNA Technologies) targeting the nucleoside monophosphate kinase *nmpk* gene (nmpk-F: 5′-TCCTTTGCCAACACCAGGAG-3′; nmpk-R: 5′-AATCCTGCCGGAGTTATGCC-3′) were used to further verify *M. bovis* identity. PCR was performed using Phusion High-Fidelity DNA Polymerase (Cat# F530L, Thermo Scientific, Massachusetts, United States) with cycle conditions of 98°C for 30 seconds, 35 cycles at 98°C for 10 seconds, 65°C for 30 seconds, 72°C for 30 seconds, and a final extension of 72°C for 10 minutes. PCR products were visualized with agarose gel electrophoresis using a GelDoc imaging system (Cat# 10000076955, Bio-Rad Laboratories, California, United States) to verify band sizes and subsequently cleaned using ExoSAP-IT Express (Cat# 75001.200.UL, Applied Biosystems, Massachusetts, United States) for Sanger sequencing with nmpk-F, 27F, and 1492R. Finally, BLAST using the NCBI *nt* database was used to verify identity of *M. bovis*.

Prior to library preparation, samples were normalized to starting concentrations of 30 ng per sample. Samples were prepared using transposome-mediated reaction Illumina Tagmentation (Illumina, California, United States, Cat # 20060059). Libraries were amplified, indexed, and subsequently cleaned. Sizes of libraries were validated using the Agilent 4150 TapeStation System (Agilent, California, United States, Cat # G2992AA). Quantification of libraries was performed using both the Qubit 4 Fluorometer (Thermo Fisher Scientific, Waltham, MA, Cat # Q33238) and NEB Illumina Library Quantification qPCR Kit (Illumina, California, United States, Cat # E7630S). Libraries were then diluted to 4 nM and pooled to prepare for sequencing with the Illumina MiSeq System (Illumina, California, United States, Cat # SY-410-1003) using v2 (2 × 250) chemistry (Illumina, California, United States, Cat # MS-102-2002). MLST sequence types were identified using mlst (github.com/tseemann/mlst) in conjunction with the PubMLST website (https://pubmlst.org/) developed in reference (19) at the University of Oxford.

In total, 24 isolates were used in this study. Both laboratory isolates and samples received from Cornell University (*n* = 17) and KPU (*n* = 7) were only available as they were successfully isolated or confirmed positive during routine procedures carried out by their respective research groups. Consequently, isolates and samples did not arrive at our laboratory in a single shipment. We collected isolates and samples over several months until we reached a sufficient number to draw meaningful conclusions, and additional shipments did not alter our results and conclusions.

### Culture procedures

**Fig 1:**
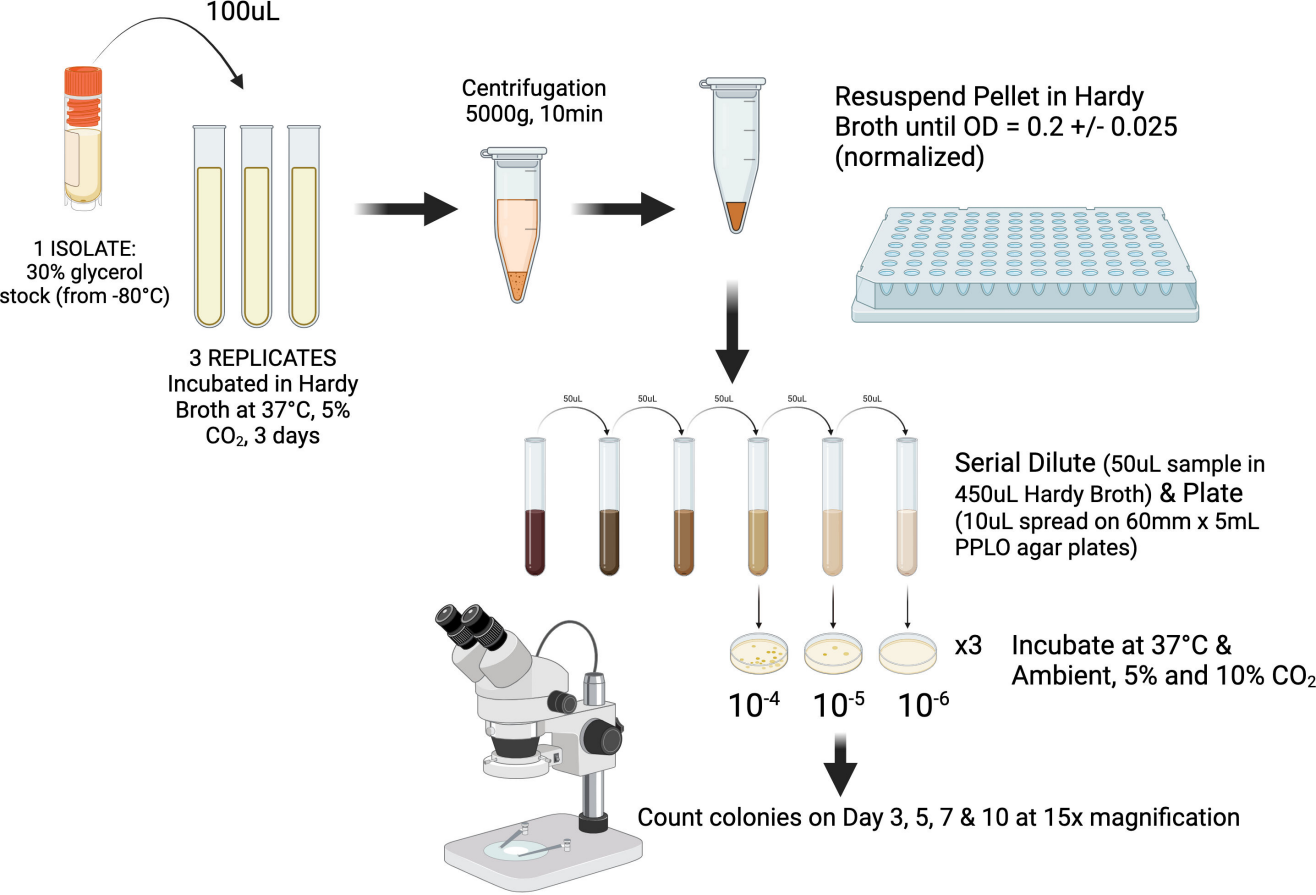
Outline of experimental design to assess impacts of ambient air, 5% CO_2_, or 10% CO_2_ on bacterial growth of 24 *Mycoplasma* spp. isolates cultured in triplicates per treatment. “Created with BioRender.com.”

PPLO agar was produced in-house, following a formulation used by Royal GD (Deventer, the Netherlands; Supplementary Materials A), based on NMC guidelines and the CVM. Agar was not supplemented with dextrose and free DNA as specified in NMC, but not in CVM. For each CO_2_ concentration and each isolate, 100 µL of one glycerol stock from each strain was inoculated in triplicate into 4.0 mL of Mycoplasma Broth (Cat# R102, Hardy Diagnostics, Santa Maria, CA). A negative control broth culture was also prepared. Adapting procedures outlined in reference (11) after 3 days of incubation at 37°C and 5% CO_2_, 1–2 mL of each triplicate was centrifuged at 5,000× *g* for 10 minutes to obtain a cell pellet, which was resuspended in PPLO Selective Broth to achieve a standardized OD_540_ of 0.2 ± 0.05. Triplicates were serially diluted, and 10 µL of the 10^−4^, 10^−5^, and 10^−6^ dilutions was spread onto 60-mm PPLO plates before incubation in ambient air, 5% CO_2_, or 10% CO_2_ for 10 days (Fig. 1). A negative control plate was prepared for each CO_2_ concentration. The CO_2_ concentration and temperature were monitored using the visual display on a Heracell 150i incubator (Thermo Fisher Scientific, Waltham, MA) with an error rate of ±0.3% for CO_2_ concentrations. Colonies, characterized by a “fried egg” appearance, were counted for all plates using a stereomicroscope (15× magnification) after 3, 5, 7, and 10 days of incubation.

### Statistical analyses

Colony-forming units (cfu) per milliliters per isolate were calculated for all plates within a countable range (25–250 colonies) (20–22). A log-10 transformation was applied to these calculated values to ensure a normal distribution. Subsequently, mean log_10_ cfu/mL was determined for each isolate on each counting day (3, 5, 7, and 10 days), based on available data from the triplicates. At each day of incubation (3, 5, 7, and 10 days), a linear regression model was employed to compare the mean log_10_ cfu/mL between CO_2_ conditions (four models total).

Bacterial growth within each triplicate was characterized as either observable (number of counted colonies > 1) or non-observable (number of counted colonies = 0). Subsequently, bacterial growth for each isolate was defined as requiring at least one of the triplicates to exhibit observable growth. For each of the three dilution ranges separately, the observable growth across isolates was compared on each counting day (3, 5, 7, and 10 days) for the three CO_2_ conditions (ambient air, 5% CO_2_, and 10% CO_2_), using a logistic regression model.

Finally, a sensitivity analysis was conducted, employing two additional methods to ascertain bacterial growth for each isolate. In the first method, bacterial growth was defined as requiring ≥2 triplicates to exhibit observable growth. In the second method, bacterial growth was defined as necessitating observable growth in all three triplicates.

Statistical significance was determined at *P* ≤ 0.05. All data cleaning, descriptive statistics, and analyses were performed using STATA/SE Version 16.1 (Stata Corp, College Station, TX).

## RESULTS

### Descriptive statistics

Among the 24 isolates, 20 (83%) were identified as *M. bovis* and 2 (9%) as *Mycoplasma canadense*, and there was 1 (4%) isolate of each *Mycoplasma alkalescens* and *Mycoplasma bovigenitalium*. The 24 isolates were obtained from composite milk samples (*N* = 8; 33%), 5 (21%) from milk quarter samples, 4 (17%) from joint aspirate samples, 3 (13%) from a lung sample, and 2 (8%) each from ear swab and bulk tank milk. Of the 20 *M*. *bovis* isolates, 11 (55%) were obtained from milk samples. The remaining nine isolates (45%) were obtained from ear swabs (*N* = 2), joint aspirate (*N* = 4), and lung tissue (*N* = 3). The total 24 isolates originated from samples collected on at least 17 unique farms. The characteristics of the 24 isolates are presented in Table 1.

**TABLE 1:** Isolate characteristics (farm, sample type, *Mycoplasma* spp., and sequencing type) of the 24 isolates^a,b^

Isolate	Farm	Sample	Species	Sequencing type
1855	5	Joint aspirate	*Mycoplasma bovis*	69
18Y-3	18	Bulk tank milk	*Mycoplasma bovis*	60
18Y-4	19	Bulk tank milk	*Mycoplasma bovis*	60
3596	13	Lung	*Mycoplasma bovis*	27
3698	12	Lung	*Mycoplasma bovis*	–
3699	4	Lung	*Mycoplasma bovis*	69
3745	7	Milk composite	*Mycoplasma bovis*	–
3752	9	Milk composite	*Mycoplasma bovis*	45
3757	2	Milk quarter	*Mycoplasma bovis*	–
3771	18	Milk quarter	*Mycoplasma bovis*	–
3775	17	Milk quarter	*Mycoplasma canadense*	–
3778	1	Milk composite	*Mycoplasma bovis*	45
3890	15	Milk composite	*Mycoplasma bovis*	–
3891	18	Milk composite	*Mycoplasma bovigenitalium*	
3893	14	Milk composite	*Mycoplasma alkalescens*	–
3894	11	Milk quarter	*Mycoplasma bovis*	–
3895	8	Milk quarter	*Mycoplasma bovis*	60
3896	16	Milk composite	*Mycoplasma bovis*	–
3898	17	Milk composite	*Mycoplasma canadense*	–
ES41223_3	NA	Ear swab	*Mycoplasma bovis*	–
JF41223_1	NA	Joint aspirate	*Mycoplasma bovis*	65
JF41223_4	NA	Joint aspirate	*Mycoplasma bovis*	65
KS41223_1	NA	Joint aspirate	*Mycoplasma bovis*	65
RE41223_1	NA	Ear swab	*Mycoplasma bovis*	65

^*a*^ NA = Not Available

^*b*^ - = MLST type could not be identified

### Colony-forming units per milliliters

In general, the raw colony counts displayed an expected 10-fold reduction across the dilutions (Supplementary Materials B). Median colony-forming units per milliliter per dilution were calculated for each isolate based on the available colony count. Cultures with <25 or >250 colonies were not included due to unquantified counts (no growth or too numerous to count). Table 2 presents median colony-forming units per milliliter data. However, it should not be interpreted as a growth curve due to the methodology employed. Growth (cfu/mL) estimation was conducted per dilution, and plates deemed too numerous to count were excluded from this estimation. Therefore, no statistical tests were deployed. Median colony-forming units per milliliter estimated in dilution 10^−4^ after 3 days of incubation in ambient air, 5% CO_2_, or 10% CO_2_, were 7.4 * 10^3^, 1.5 * 10^4^, and 1.3 * 10^4^, respectively. After 10 days of incubation, these values were 1.3 * 10^4^, 1.5 * 10^4^, and 1.8 * 10^4^ cfu/mL. For dilution 10^−5^, median colony-forming units per milliliter after 3 days of incubation in ambient air, 5% CO_2_, or 10% CO_2_ were 9.4 * 10^4^, 6.3 * 10^4^, and 1.1 * 10^5^, respectively. After 10 days of incubation, these values were 9.7 * 10^4^, 9.2 * 10^4^, and 1.1 * 10^5^ cfu/mL. For dilution 10^−6^, median colony-forming units per milliliter after 3 days of incubation in ambient air, 5% CO_2_, or 10% CO_2_ were no growth, 2.9 * 10^5^, and 3.0 * 10^5^, respectively. After 10 days of incubation, these values were 6.8 * 10^5^, 3.5 * 10^5^, and 3.0 * 10^5^ cfu/mL (Table 2).

**TABLE 2:** Median and interquartile range of the cfu/mL for countable (>25 and <250 colonies) plates of *Mycoplasma* spp. isolates after 3, 5, 7, and 10 days of incubation in ambient air, 5% CO_2_, or 10% CO_2_^*a*,*b*^

Days incubated	Ambient air			5% CO_2_			10% CO_2_		
	Median	25% quartile	75% quartile	Median	25% quartile	75% quartile	Median	25% quartile	75% quartile
D 10^−4^									
3	7.4 * 10^3^	5.6 * 10^3^	1.3 * 10^4^	1.5 * 10^4^	6.9 * 10^3^	1.7 * 10^4^	1.3 * 10^4^	8.9 * 10^3^	1.5 * 10^4^
5	1.6 * 10^4^	1.1 * 10^4^	1.9 * 10^4^	1.7 * 10^4^	1.6 * 10^4^	1.9 * 10^4^	2.2 * 10^4^	1.6 * 10^4^	2.3 * 10^4^
7	1.1 * 10^4^	5.9 * 10^3^	1.7 * 10^4^	1.6 * 10^4^	1.4 * 10^4^	1.7 * 10^4^	1.5 * 10^4^	1.4 * 10^4^	2.2 * 10^4^
10	1.3 * 10^4^	6.8 * 10^3^	1.5 * 10^4^	1.5 * 10^4^	7.7 * 10^3^	1.8 * 10^4^	1.8 * 10^4^	1.5 * 10^4^	2.3 * 10^4^
D 10^−5^									
3	9.4 * 10^4^	6.5 * 10^4^	9.8 * 10^4^	6.3 * 10^4^	4.0 * 10^4^	1.1 * 10^5^	1.1 * 10^5^	7.0 * 10^4^	1.3 * 10^5^
5	6.4 * 10^4^	4.3 * 10^4^	1.0 * 10^5^	1.2 * 10^5^	8.2 * 10^4^	1.6 * 10^5^	1.3 * 10^5^	7.1 * 10^4^	1.4 * 10^5^
7	8.9 * 10^4^	5.5 * 10^4^	1.1 * 10^5^	9.4 * 10^4^	8.3 * 10^4^	1.5 * 10^5^	1.1 * 10^5^	7.5 * 10^4^	1.4 * 10^5^
10	9.7 * 10^4^	5.1 * 10^4^	1.1 * 10^5^	9.1 * 10^4^	7.9 * 10^4^	1.3 * 10^5^	1.1 * 10^5^	8.1 * 10^4^	1.4 * 10^5^
D 10^−6^									
3	NG	NG	NG	2.9 * 10^5^	2.6 * 10^5^	3.1 * 10^5^	3.0 * 10^5^	2.9 * 10^5^	3.0 * 10^5^
5	6.6 * 10^5^	6.6 * 10^5^	6.6 * 10^5^	3.2 * 10^5^	3.0 * 10^5^	3.8 * 10^5^	3.0 * 10^5^	2.7 * 10^5^	3.4 * 10^5^
7	6.9 * 10^5^	6.9 * 10^5^	6.9 * 10^5^	3.5 * 10^5^	3.2 * 10^5^	3.6 * 10^5^	2.7 * 10^5^	3.1 * 10^5^	3.3 * 10^5^
10	6.8 * 10^5^	6.8 * 10^5^	6.8 * 10^5^	3.5 * 10^5^	3.4 * 10^5^	3.7 * 10^5^	3.0 * 10^5^	2.7 * 10^5^	3.9 * 10^5^

^*a*^ Number of observations used for estimating cfu/mL change per incubation day within each dilution due to no quantified counts for plates that were too numerous to count.

^*b*^ D = dilution; NG = no growth.

**Fig 2:**
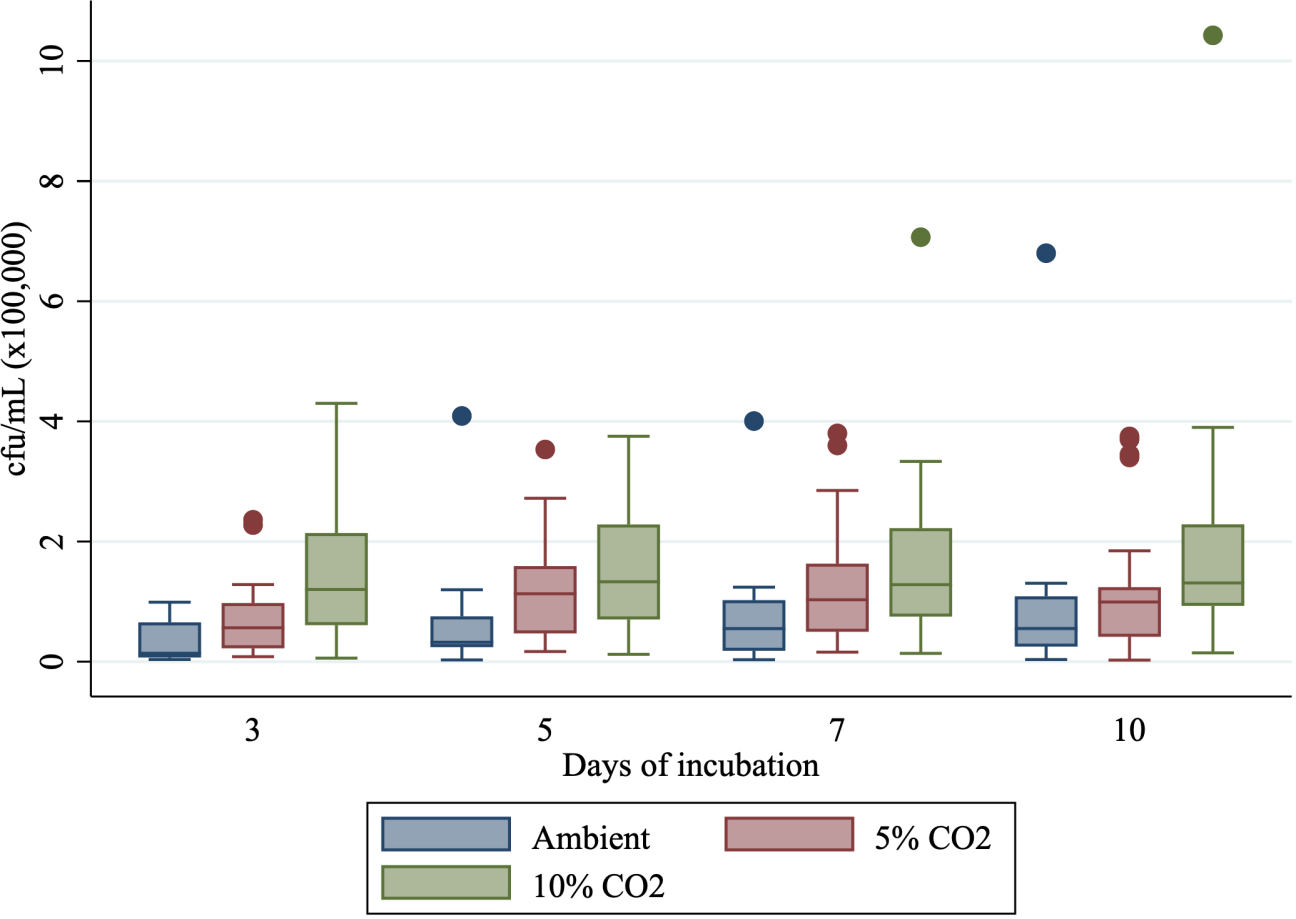
Growth (cfu/mL) for isolates incubated in ambient air, 5% CO_2_, or 10% CO_2_ after 3, 5, 7, or 10 days of incubation.

When using all dilutions to obtain an estimated cfu/mL per isolate, there was less pronounced variation in growth (cfu/mL) when incubated in ambient air compared with 5% CO_2_ or 10% CO_2_ (Fig. 2). Median colony-forming unit per milliliter for 10% CO_2_ was consistent throughout the incubation period, being 1.2 * 10^5^ on day 3 to 1.3 * 10^5^ on days 5, 7, and 10. Median colony-forming units per milliliter for 5% CO_2_ increased from 5.6 * 10^4^ on day 3 to 1.1 * 10^5^, 1.0 * 10^5^, and 1.0 * 10^5^ on days 5, 7, and 10. Median colony-forming units per milliliter for ambient conditions increased from 1.4 * 10^4^ on 3 days of incubation to 3.2 * 10^4^, 5.5 * 10^4^, and 5.5 * 10^4^ on days 5, 7, and 10 of incubation.

The overall bacterial growth of isolates, as determined by log_10_ cfu/mL to ensure normality, based on results of all dilutions, was lower after 3, 5, and 7 days of incubation when cultured in ambient air compared with isolates incubated in 5% or 10% CO_2_ (*P =* 0.04, *P* = 0.01, and *P* = 0.03 or *P <* 0.001, *P* < 0.01, *P* < 0.01, respectively). After 10 days of incubation, log_10_ cfu/mL did not differ between isolates incubated in ambient air and 5% CO_2_ (*P =* 0.22), whereas log_10_ cfu/mL was lower for ambient air compared with 10% CO_2_ (*P <* 0.01).

**Fig 3:**
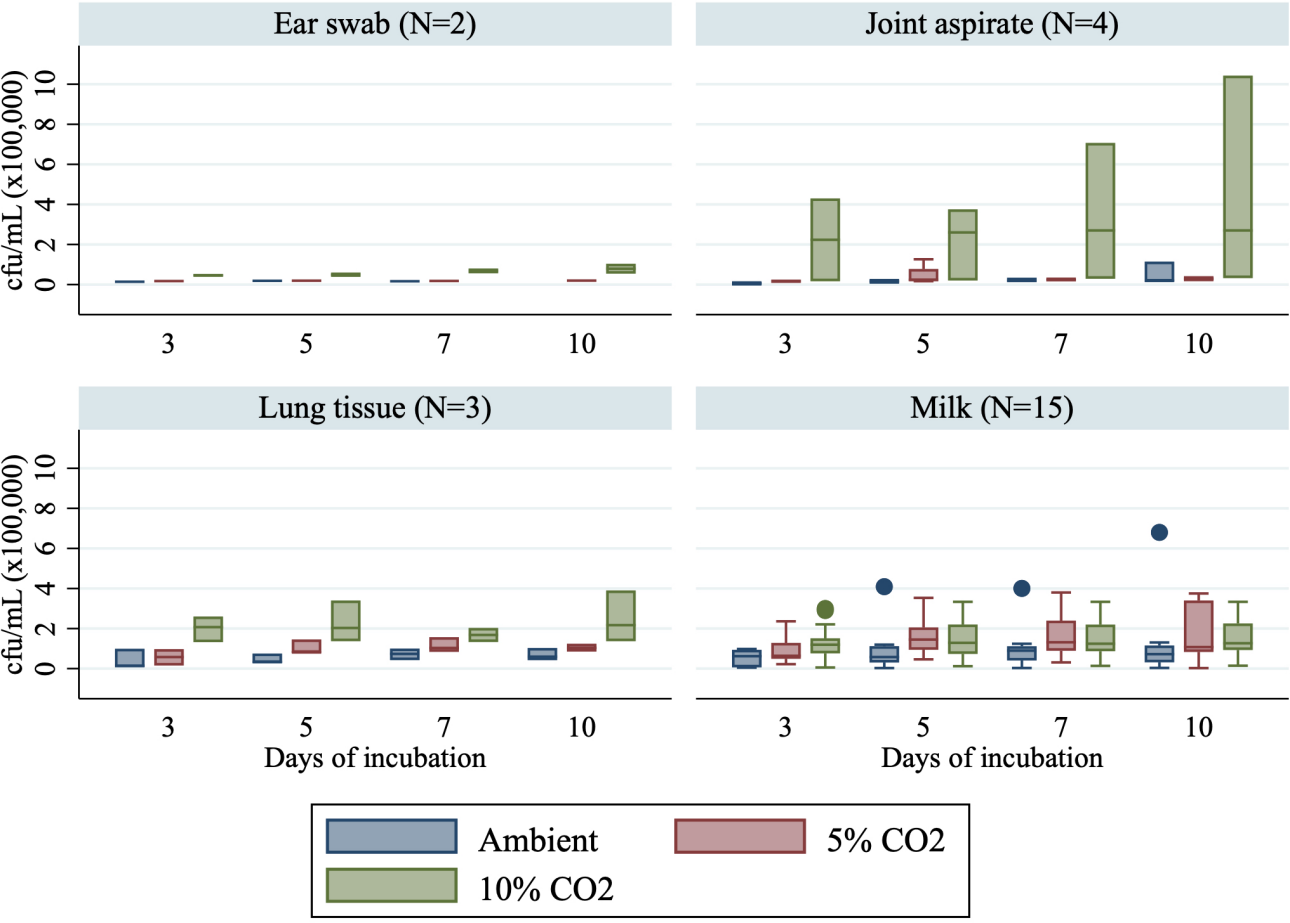
Growth (cfu/mL) for isolates obtained in ear swabs, joint aspirate, lung tissue, and milk samples incubated in ambient air, 5% CO_2_, or 10% CO_2_ after 3, 5, 7, or 10 days of incubation.

**Fig 4:**
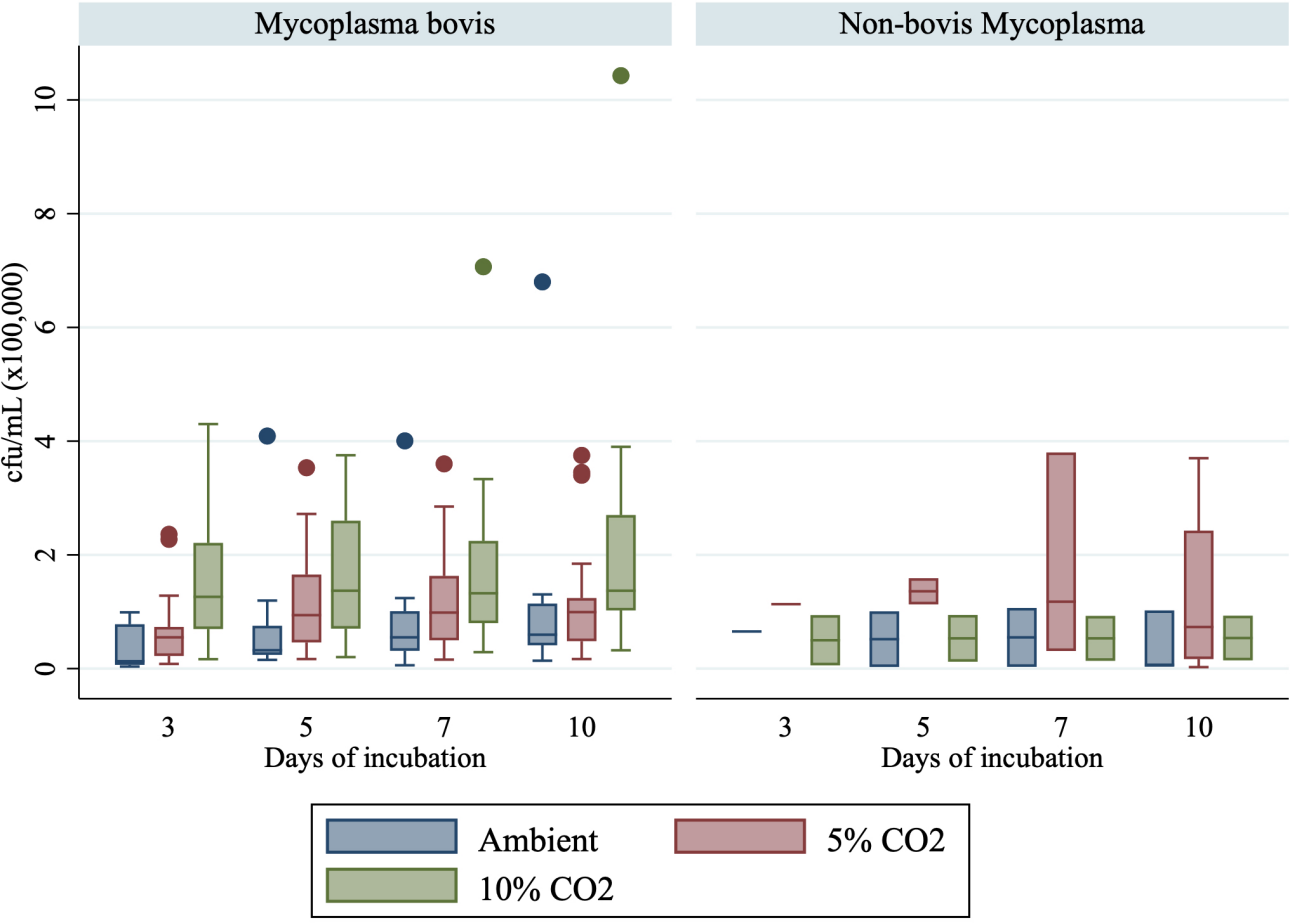
Growth (cfu/mL) for isolates identified as *Mycoplasma bovis* (*N* = 20) and non-*bovis Mycoplasma* (*N* = 4) incubated in ambient air, 5% CO_2_, or 10% CO_2_ after 3, 5, 7, or 10 days of incubation.

Estimation of cfu/mL was conducted for isolates obtained from the four distinct sample types: ear swabs, joint aspirate, lung tissue, and milk sample. Median cfu/mL in milk samples followed a similar pattern as previously described (Fig. 3). For isolates obtained from the four joint aspirate samples, median cfu/mL was consistently higher when incubated in 10% CO_2_ (2.2, 2.6, 2.7, and 2.7 * 10^5^ cfu/mL on days 3, 5, 7, and 10) compared with 5% CO_2_ (1.7, 2.3, 2.9, and 2.9 * 10^4^ cfu/mL on days 3, 5, 7, and 10) and ambient air (5.7 * 10^3^, 1.6, 1.8, and 1.8 * 10^4^ cfu/mL on days 3, 5, 7, and 10) (Fig. 3). Median colony-forming units per milliliter were higher for non-*bovis* isolates when incubated in 5% CO_2_ compared with 10% CO_2_ and ambient air (Fig. 4).

### Growth detection

There were no differences in detection of observable growth (absence of growth vs. presence of at least one colony) among isolates incubated in ambient air, 5% CO_2_, and 10% CO_2_ for all three dilutions on day 10 (Table 3). Regarding dilution 10^−4^, there were no differences in detection of observable growth on any incubation days. For dilution 10^−5^, a difference in the detection of observable growth was noted between ambient air and 5% CO_2_ or 10% CO_2_ on 3 days of incubation (*P* = 0.02; *P* = 0.02). For dilution 10^−6^, differences in the detection of observable growth emerged between ambient air and 5% CO_2_ and 10% CO_2_ on 3 days of incubation (*P* = 0.04; *P* < 0.01) and between ambient air and 10% CO_2_ on 7 days of incubation (*P* = 0.04).

**TABLE 3:** Observable growth (dilution 10^−4^, 10^−5^, and 10^−6^) of 24 *Mycoplasma* spp. isolates after 3, 5, 7, and 10 days of incubation in ambient air, 5% CO_2_, or 10% CO_2_

Days incubated	Ambient air	5% CO_2_	10% CO_2_
	No. (%)	No. (%)	No. (%)
Dilution 10^−4^			
3	18 (75)	21 (88)	23 (96)
5	22 (92)	24 (100)	23 (96)
7	23 (96)	24 (100)	23 (96)
10	24 (100)	24 (100)	23 (96)
Dilution 10^−5^			
3	13 (54)	21 (88)	21 (88)
5	18 (75)	24 (100)	23 (96)
7	20 (83)	24 (100)	23 (96)
10	24 (100)	24 (100)	23 (96)
Dilution 10^−6^			
3	11 (46)	18 (75)	21 (88)
5	17 (71)	22 (92)	22 (92)
7	17 (71)	22 (92)	23 (96)
10	22 (92)	23 (96)	23 (96)

For method 2 (requiring ≥2 of the triplicates to exhibit growth), observable growth in dilutions 10^−4^, 10^−5^, and 10^−6^ after 3 days of incubation was lower using ambient air compared with 5% CO_2_ and 10% CO_2_ (day 3 in 10^−4^: *P =* 0.001; *P =* 0.001; day 3 in 10^−5^: *P <* 0.001; *P <* 0.001; day 3 in 10^−6^: *P <* 0.01; *P <* 0.001) (Supplementary Materials C). However, there were no significant differences at 5, 7, or 10 days of incubation.

For method 3 (requiring all triplicates to exhibit growth), observable growth in dilutions 10^−4^, 10^−5^, and 10^−6^ after 3 days of incubation was lower when using ambient air compared with 5% CO_2_ or 10% CO_2_ on 3 days of incubation (day 3 in 10^−4^: *P <* 0.001; *P <* 0.001; day 3 in 10^−5^: *P =* 0.001; *P <* 0.001; day 3 in 10^−6^: *P =* 0.03; *P <* 0.001) (Supplementary Materials C). This difference persisted after 5 days of incubation for dilution 10^−6^ for ambient air compared with 5% CO_2_ or 10% CO_2_ (*P =* 0.01, *P <* 0.01, respectively). However, there were no significant differences on 7 or 10 days of incubation.

## DISCUSSION

Current recommendations for culturing *Mycoplasma* spp. from laboratory isolates obtained from dairy cow samples were systematically examined to determine the impacts of varying percentages of supplemented CO_2_ on growth. Firstly, consistently lower log_10_ cfu/mL were observed for isolates incubated in ambient air compared with those in 5% CO_2_ or 10% CO_2_ across most time points. Additionally, the variability of colony-forming units per milliliter was markedly lower in isolates incubated in ambient conditions, displaying more homogeneity than isolates incubated under 5% and especially 10% CO_2_ (Fig. 2). This suggests that *Mycoplasma* spp. exhibit sensitivity to supplemental CO_2_ during incubation. Established knowledge suggests that many anaerobic bacteria experience enhanced growth in the presence of small amounts of supplementary CO_2_ (23). Notable size differences were identified during counting, with colonies incubated in ambient air being smaller than those incubated in 5% CO_2_ or 10% CO_2_ (Supplementary Materials D). It is acknowledged that counting such small colonies may result in varied counts from day to day. Consequently, this may lead to an underestimation of the number of colonies counted in ambient cultures compared with those in 5% CO_2_ or 10% CO_2_ and could explain why the estimated colony-forming unit per milliliter was consistently lower. Unfortunately, time restrictions prevented measurements of colony size. Therefore, accurately comparing these measures between CO_2_ concentration was not feasible.

Secondly, there was a difference in observable growth at 3 days of incubation, revealing a lower detection rate for isolates incubated in ambient air when compared with those in 5% CO_2_ or 10% CO_2_. However, no differences were observed at 7 and 10 days of incubation across all tested dilution ranges. This suggests that, adhering to the recommended incubation period of at least 7 days by the NMC, there was no significant divergence in detection rates among cultures incubated in ambient, 5% CO_2_, or 10% CO_2_. These results were in line with an earlier study where no significant differences in growth detection in laboratory isolates were reported for *Mycoplasma* spp. cultures incubated in candle jars, 5% CO_2_, or 10% CO_2_ (11). Increased growth rates on days 3 and 5 of incubation could be supported by the fact that elevated CO_2_ concentrations (5%) are present in mammalian tissues (24), possibly favoring these conditions over the absence of supplemental CO_2_. However, since there was no difference in detection on 10 days of incubation, perhaps, other factors have a more impactful role in *Mycoplasma* spp. growth, e.g., glycerol and cholesterol availability (25), cell membrane components, and potentially pH (26). Additionally, because the CO_2_ concentration has been known to affect the minimum inhibitory concentration of other bacteria (27, 28), it would be of interest to perform a similar study in the future to elucidate if there would be any variation in *Mycoplasma* MIC data as a result of chosen incubation conditions.

It is essential to emphasize that these findings are derived from pure isolates, rather than original specimens, such as positive milk. Additionally, these results are primarily relevant to *M. bovis*, as obtaining meaningful data from isolates of other species was challenging due to their limited availability and the high prevalence of *M. bovis* species. Consequently, it was not feasible to accurately assess species and sample type differences. Nonetheless, given that *M. bovis* is the most prevalent *Mycoplasma* spp. in dairy herds globally (29), our findings remain of significant importance. Distinctive properties among bovine *Mycoplasma* spp. have been elucidated, such as differences in the ability to obtain acids from glucose, arginine catabolism, film and spot production, and aerobic tetrazolium reduction, which have been described among *Mycoplasma bovirhinis*, *Mycoplasma dispar*, *M. alkalescens*, *Mycoplasma arginini*, *M. canadense*, *M. bovigenitalium*, and *M. bovis* (13) The extent to which these differences impact diagnostic culture performance related to CO_2_ supplementation remains unclear. This study’s findings regarding growth detection remained unchanged even when excluding the four non-*bovis Mycoplasma* species from the analysis (results not shown). Additionally, when considering the various sample types, there is a suggestion that joint aspirate may respond differently to CO_2_ supplementation than milk samples. In our study, joint aspirate samples demonstrated the highest colony-forming units per milliliter when incubated in 10% CO_2_. However, it is important to note that only four samples were included, and therefore, more extensive research is warranted before a robust conclusion can be drawn, and results can be generalized to other *Mycoplasma* species and sample types.

On average, a *M. bovis* culture-positive individual milk sample contains between 5 * 10^4^ and 8 * 10^8^ cfu/mL as described in reference (30). For cows in the prodromal stage of clinical and subclinical mastitis, this is estimated to be 10^3^–10^6^ cfu/mL (31), and for cows with clinical mastitis, the estimated rates of *Mycoplasma* spp. excretion increase to 10^5^–10^8^ cfu/mL (32). In this study, OD standardization and a dilution range were applied to mimic low-positive *Mycoplasma* spp. samples and test whether supplementary CO_2_ had an impact on low-positive samples. In our dilution ranges (10^−4^, 10^−5^, and 10^−6^), median colony-forming unit per milliliter for all treatments on each of the incubation days was estimated to be between 10^4^ and 10^5^, respectively, mimicking samples of cows with subclinical mastitis.

The lowest colony-forming unit per milliliter in our study was estimated to be 3.6 * 10^3^ (ambient air, 3 days of incubation) and the highest to be 2.0 * 10^6^ (10% CO_2_, 10 days of incubation). Lowest colony-forming units per milliliter were mainly observed on 3 days of incubation and highest on 10 days of incubation which was in line with the 0.17 log_10_ cfu/mL increase when incubated for 7 days, as described in reference (11). However, in this study, a clear increase in log_10_ cfu/mL was only observed under ambient conditions, extending up to 7 days, whereas the log_10_ cfu/mL for 5% CO_2_ or 10% CO_2_ remained consistent throughout the incubation period. This suggests that proliferation of *Mycoplasma* spp. colonies occurred within the initial 7 days when cultured under ambient conditions, necessitating extended incubation intervals.

Various types of agars are widely used, with PPLO and *Mycoplasma* agar being two common variants (5). In this study, the PPLO agar formulation deviated slightly from the NMC formulation, utilizing ampicillin instead of penicillin and omitting dextrose and free DNA, as outlined in CVM (16). Ampicillin, a semi-synthetic penicillin, demonstrates efficacy against both Gram-negative and Gram-positive organisms, in contrast to penicillin that targets only Gram-positive organisms (33). Consequently, the anticipated impact of ampicillin versus penicillin on colony growth was deemed negligible. Dextrose (omitted in our study), typically added as an energy source to promote bacterial growth, may not have a significant role for *M. bovis* and *M. agalactiae* either. These species are known to eschew glucose fermentation and prefer non-sugar carbon sources, relying instead on organic acids, lactate, and pyruvate for their energy needs (34, 35). PPLO agar is largely based on peptones, which are water-soluble protein hydrolysates (36), and yeast, containing >10% carbohydrates (37). *Mycoplasma* spp. largely utilize (forms of) carbohydrates as the energy source for growth as specified above. Moreover, horse serum contains cholesterol, protein, and glucose. Despite the tendency to avoid glucose fermentation, it remains a viable option as an alternative energy source for promoting growth. Therefore, potential differences in growth effect due to utilizing a slight modification in agar preparation are deemed negligible.

Isolates obtained for this study were resuscitated by incubating them for 3 days at 37°C in 5% CO_2_ before the start of the experiment to ensure live cultures. This selection criteria bias our study toward *Mycoplasma* isolates that are proven to grow when incubated in 5% CO_2_. Our aim was to compare ambient conditions to CO_2_ supplementation, and therefore, it was chosen not to obtain isolates from protocols that deviated from the NMC to limit bias toward non-conventional ambient conditions.

A similar study was conducted in 2018, also aimed to evaluate growth of *Mycoplasma* spp. under varying conditions, including incubation in candle jars, 5% CO_2_, and 10% CO_2_ (11). It was hypothesized that the range of CO_2_ might be broader than what was used in this study. While no differences in growth detection rate were observed after 7 days of incubation, detection rates were higher for 5% CO_2_ or 10% CO_2_ on 3 or 5 days of incubation. These findings suggest potential implications for enhancing efficiency in diagnostic laboratories by supplementing CO_2_, potentially reducing turnaround times, and improving economics. Conversely, the ability to culture *Mycoplasma* spp. without supplemental CO_2_ presents opportunities for veterinary practices or larger dairy farms to conduct in-house or on-farm culturing. However, validating the results of this standardized laboratory experiment warrants a more comprehensive study to better reflect real-world scenarios. Such a study should include multiple *Mycoplasma* species and field samples collected from diverse individual cows and farms, expressing a broad spectrum of clinical and subclinical symptoms. Additionally, although a growth curve was not established in this study, which could have provided valuable insights, standardizing OD measurements for *Mycoplasma* broth cultures poses significant challenges due to their insufficient turbidity. As a result, many diagnostic laboratories lack a standardized OD protocol. In our study, we successfully employed the OD methodology described after centrifuging our samples.

Nevertheless, this study serves a proof-of-concept for culturing *Mycoplasma* spp. under ambient air conditions.

### Conclusions

Lower bacterial growth (log_10_ cfu/mL) was detected in *Mycoplasma* spp. cultures after 3, 5, and 7 days of incubation in ambient air compared with isolates incubated in 5% CO_2_ or 10% CO_2_. This difference retained significance for log_10_ cfu/mL on 10 days of incubation between ambient air and 10% CO_2_. However, no significant differences in growth detection rates of *Mycoplasma* spp. cultured under ambient air, 5% CO_2_, or 10% CO_2_ were observed after 7 days of incubation. Consequently, from a diagnostic perspective, we concluded that *Mycoplasma* spp. and, particularly, *M. bovis* demonstrate growth even in the absence of supplemental CO_2_ but may require a longer incubation. Therefore, we recommend expanding the utilization of CO_2_ in *M. bovis* culture procedures to a range including ambient air to 5% CO_2_ while maintaining incubation for a duration of 7–10 days, because 10% CO_2_ was inferior to 5% CO_2_ tension.

## SUPPLEMENTAL MATERIAL

Supplemental material spectrum.00946-24-s0001.docx Tables S1 to S6; Fig. S1.

Supplemental data spectrum.00946-24-s0002.xlsx Raw colony count.

ASM does not own the copyrights to Supplemental Material that may be linked to, or accessed through, an article. The authors have granted ASM a non-exclusive, world-wide license to publish the Supplemental Material files. Please contact the corresponding author directly for reuse.
