# Supplementary material for: Impact of carbon dioxide concentrations on laboratory sensitivity of Mycoplasma species isolated from dairy cows
Source: Microbiol Spectr. 2024 Aug 20;12(10):e00946-24. doi: 10.1128/spectrum.00946-24 (PMC11448075; doi:10.1128/spectrum.00946-24)
Supplement: Supplemental material — Tables S1 to S6; Fig. S1. [file spectrum.00946-24-s0001.docx]

**Supplementary Materials**

1. **Agar preparation**

Solutions of 1% (w/v) Thallium acetate (Cat#T8266, Sigma) and 10% (w/v) Yeast Extract (Cat#70161, Millipore, Sigma) were prepared and sterilized by filtration and autoclaving at 121ºC for 15 minutes, respectively. BD Difco PPLO Agar (Cat# 241210) was prepared as directed on the label, autoclaved at 121ºC for 15 minutes and left to acclimate to 55ºC before the addition of: pre-sterilized Horse Serum (Cat#16050-122, Gibco), 100mg/mL Ampicillin (Cat#A5354, Sigma), 10% Yeast Extract solution, and 1% Thallium Acetate solution to the final concentrations of:

**Per Liter of PPLO Agar**

| Horse serum | 197.49 mL |
| --- | --- |
| Yeast extract | 9.87 g |
| Thallium acetate | 85.90 mg |
| Ampicillin | 197.49 mg |

1. **Sensitivity Analysis**

**Alternative method 1: observable growth defined as growth detected on at least two of the triplicates.**

**Table S1.** Observable growth (dilution 10^-4^) of 24 *Mycoplasma* spp. isolates after 3, 5, 7 and 10 days of incubation in ambient air, 5 and 10% CO_2_

| Days incubated | Ambient air |  | 5% CO_2_ |  | 10% CO_2_ |
| --- | --- | --- | --- | --- | --- |
|  | No. (%) |  | No. (%) |  | No. (%) |
| 3 | 9 (38) |  | 21 (88) |  | 22 (92) |
| 5 | 21 (88) |  | 24 (100) |  | 23 (96) |
| 7 | 23 (96) |  | 24 (100) |  | 23 (96) |
| 10 | 24 (100) |  | 24 (100) |  | 23 (96) |

**Table S2.** Observable growth (dilution 10^-5^) of 24 *Mycoplasma* spp. isolates after 3, 5, 7 and 10 days of incubation in ambient air, 5 and 10% CO_2_

| Days incubated | Ambient air |  | 5% CO_2_ |  | 10% CO_2_ |  |
| --- | --- | --- | --- | --- | --- | --- |
|  | No. (%) |  | No. (%) |  | No. (%) | |
| 3 | 5 (21) |  | 18 (75) |  | 21 (88) | |
| 5 | 18 (75) |  | 22 (92) |  | 22 (92) | |
| 7 | 18 (75) |  | 24 (100) |  | 22 (92) | |
| 10 | 22 (92) |  | 24 (100) |  | 22 (92) | |

**Table S3.** Observable growth (dilution 10^-6^) of 24 *Mycoplasma* spp. isolates after 3, 5, 7 and 10 days of incubation in ambient air, 5 and 10% CO_2_

| Days incubated | Ambient air |  | 5% CO_2_ |  | | 10% CO_2_ | |  |
| --- | --- | --- | --- | --- | --- | --- | --- | --- |
|  | No. (%) |  | No. (%) | |  | | No. (%) | |
| 3 | 3 (13) |  | 13 (54) | |  | | 19 (79) | |
| 5 | 16 (67) |  | 19 (79) | |  | | 21 (88) | |
| 7 | 17 (71) |  | 20 (83) | |  | | 21 (88) | |
| 10 | 19 (79) |  | 20 (83) | |  | | 21 (88) | |

**Alternative method 2: observable growth defined as growth detected all three triplicates.**

**Table S4.** Observable growth (dilution 10^-4^) of 24 *Mycoplasma* spp. isolates after 3, 5, 7 and 10 days of incubation in ambient air, 5% and 10% CO_2_

|  | Ambient air |  | 5% CO_2_ |  | 10% CO_2_ |
| --- | --- | --- | --- | --- | --- |
|  | No. (%) |  | No. (%) |  | No. (%) |
| Day 3 | 6 (25) |  | 19 (79) |  | 22 (92) |
| Day 5 | 18 (75) |  | 22 (92) |  | 23 (96) |
| Day 7 | 22 (92) |  | 22 (92) |  | 23 (96) |
| Day 10 | 24 (100) |  | 22 (92) |  | 23 (96) |

**Table S5.** Observable growth (dilution 10^-5^) of 24 *Mycoplasma* spp. isolates after 3, 5, 7 and 10 days of incubation in ambient air, 5 and 10% CO_2_

|  | Ambient air |  | | 5% CO_2_ | |  | 10% CO_2_ |
| --- | --- | --- | --- | --- | --- | --- | --- |
|  | No. (%) | |  | | No. (%) |  | No. (%) |
| Day 3 | 3 (13) | |  | | 15 (63) |  | 21 (88) |
| Day 5 | 17 (71) | |  | | 19 (79) |  | 22 (92) |
| Day 7 | 18 (75) | |  | | 20 (83) |  | 22 (92) |
| Day 10 | 21 (88) | |  | | 22 (92) |  | 22 (92) |

**Table S6.** Observable growth (dilution 10^-6^) of 24 *Mycoplasma* spp. isolates after 3, 5, 7 and 10 days of incubation in ambient air, 5 and 10% CO_2_

| Days incubated | Ambient air |  | | 5% CO_2_ | |  | | 10% CO_2_ |  |
| --- | --- | --- | --- | --- | --- | --- | --- | --- | --- |
|  | No. (%) | |  | | No. (%) | |  | No. (%) | |
| 3 | 1 (4) | |  | | 8 (33) | |  | 19 (79) | |
| 5 | 8 (33) | |  | | 17 (71) | |  | 19 (79) | |
| 7 | 15 (63) | |  | | 18 (75) | |  | 21 (88) | |
| 10 | 16 (67) | |  | | 18 (75) | |  | 1. 88) | |

1. **Colony Size**

**
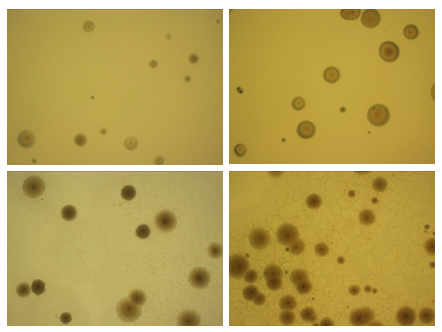
**

**D**

**C**

**B**

**A**

**Figure S1**. Colony growth of replicate 1 of a *Mycoplasma bovis* culture incubated in ambient air and 5% CO_2_ on day 3 (A - B) and day 10 (C - D) under 40x magnification (Olympus CKX53, Olympus Life Sciences).
